# Inequalities in childhood stunting: evidence from Sudan multiple indicator cluster surveys (2010–2014)

**DOI:** 10.1186/s12889-022-13145-5

**Published:** 2022-04-12

**Authors:** Bashaw Wogderes, Gebretsadik Shibre, Betregiorgis Zegeye

**Affiliations:** 1 Medical Service Team, Yeka Health Office, Addis Ababa, Ethiopia; 2 Department of Reproductive Health and Health Services Management, School of Public Health, Addis Ababa University, Addis Ababa, Ethiopia; 3 HaSET Maternal and Child Health Research Program, Shewarobit Field Office, Shewarobit, Ethiopia

**Keywords:** Stunting, Inequality, Sudan, MICS

## Abstract

**Background:**

Leaving no one behind has been an important marker of the Sustainable Development Goals. Closing the gap in malnutrition between children of different backgrounds aligns well with the tenet of this international agenda. To this end, high-quality evidence of the magnitude and trends of socioeconomic and geographic related existing inequalities in the childhood stunting among Sudanese children emanate from this study help for policy maker and planners to design and implement effective interventions to narrow down inequality.

**Methods:**

We used the World Health Organization’s (WHO) Health Equity Assessment Toolkit (HEAT) for our analysis of stunting inequality. Following standard equity analysis methods recommended by the WHO, we performed the disaggregated analysis of stunting across five equity stratifiers: Wealth, education, residence, sex, and sub-national regions. Then, we summarized stunting inequality through four measures of inequality: Difference, Ratio, Slope Index of Inequality (SII), and Relative Index of Inequality (RII). The point estimates of stunting were accompanied by 95% confidence intervals to measure the statistical significance of the findings.

**Results:**

In this study, the national average childhood stunting prevalence was increased by 4% from 2010 to 2014. The findings revealed stark inequalities in stunting in all the studied dimensions of inequality. Huge inequality has existed along the wealth quintiles. Simple difference measure for education was increased by four points and simple relative measure decreased by one point for economic status.

**Conclusions:**

Sex, residence and, geographically related inequalities remain unchanged over time, while economic status and educational inequality had seen a change by some inequality measures over the same time period.

## Introduction

Under-five child stunting represents poor linear growth and is diagnosed as a height for age less than 2 standard deviations (-2SD) from the World Health Organization (WHO) child growth standards median [1]. It is a result of poor nutrition over a long period in children under-five years [2, 3]. Most stunting cases occur in the first 1000 days following conception, and stunting which occurs during this critical period is irreversible [2, 4]. As human growth occurs during early childhood, stunting in this period has a marked effect on human development [5].

Stunting has declined globally by an estimated 40 million cases between 1980 and 2000 [6]. However, 159 million children still fail to achieve their linear growth globally [5, 7]. Research shows that stunting has been distributed unevenly globally. Low-and-Middle Income Countries (LMICs) are the especially centerpiece of stunting [8]. Three-quarters of the world’s stunted children concentrate in just two of the world’s poorest regions, Sub-Saharan Africa (SSA) and South Asia [9]. In Africa, two in every five under-five children are affected by stunting, with the highest burden in eastern region of the continent [10]. Like most countries in the eastern Africa, Sudan is one of the worst affected countries by malnutrition [11] where one in every three children under five years is stunted [12]. Furthermore, the rate of reduction in stunting has been slow in Africa [13]. Inequalities in access to basic services and the use of improper feeding practices in early childhood play a big role in the unacceptably high burden of stunting in Africa [14].

The burden of stunting in a country varies by whether or not the child lives in rural settings, is born from a non-educated and poor families, and is male or female. Evidence revealed that children living in poor households and those living in rural areas are more likely to be stunted than children from richer households and living in urban areas [15–18]. This is related to the fact that rural areas are deprived of basic intervention [12] compared with an urban settings. Studies also identified that inequality in stunting with respect to access to education is a prevailing phenomenon [19]. The child born from an uneducated family had poor nutritional status than child born from an educated family [20, 21]. A growing body of evidence showed stunting to disproportionately affect male children than female counterparts [21–23].

Stunting carries along with it numerous repercussions for the child as well as for the entire family. Over 6 million deaths occur each year worldwide due to stunting [6]. While, those who survive are at increased risk to lower educational performance and reduced earning in adulthood [7]. In resource-constrained settings, stunting causes more than 200 million children not to achieve their optimal level of cognitive development and had an impact later in adulthood on economic productivity [24]. For that matter, countries in Africa costs as high as ~ 10% of their Gross Domestic Product (GDP) due to stunting in children [25]. Malnutrition in early childhood also makes an individual more prone to communicable and non-communicable diseases later in life that indirectly become health costs of the family [26].

WHO targets the reduction of stunting by 40% by 2025 [27]. The United Nations Sustainable Development Goals (SDG) identified stunting as a key development indicator used to measure progress towards its goal to end hunger [28]. Though the SDGs want to ensure an opportunity that no child is left behind, more than one-third of 3–4-year olds living in LMICs were not on track in their cognitive and social-emotional development [15]. To achieve the above-mentioned targets, economic and social problems [25] of the member countries, including Sudan, need to be addressed. In Sudan specifically, there is a dearth of high-quality evidence on the extent of inequality in the distribution of the burden of stunting. To this end, we aim to provide policymakers and planners in Sudan with evidence of magnitude and trends of socioeconomic and geographic related inequalities in childhood stunting using the 2010–2014 Sudan demographic and health surveys. The high-quality evidence that emanate from the study will inform the design and implementation of effective interventions to narrow down, and if possible, eliminates the existing inequality in the distribution of childhood stunting across various equity stratifiers [15].

## Methods

### Data source

The source of our analysis is the offline version of the WHO HEAT software. A detailed discussion of the software has been available elsewhere [29, 30]. But in brief, the HEAT is software that enables examination and analysis of health inequalities within and between countries. The software is extremely valuable to explore the health inequality situation in more systematic detail. The HEAT software application comprises the WHO Health Equity Monitor (HEM) database [31]. The database stores data coming from Demographic and Health Survey (DHS) and Multiple Indicator Cluster Survey (MICS) conducted in many low-or-middle income countries including Mauritania. Currently, the database provides detailed inequality assessment for more than 30 Reproductive, Maternal, Newborn, and Child health indicators.

For the present study, we used the dataset derived from the two waves of the Sudan Multiple Indicator Cluster Survey (MICS) conducted in 2010 and 2014 that are found in the software. MICS was developed by UNICEF in the 1990s as an international household survey program to collect internationally comparable data on a wide range of indicators on the situation of children and women [32]. The Sudan MICS is a nationally representative survey designed to collect information on various women and child health topics such as stunting. By providing the government of Sudan with valid and up-to-date health indicators on women aged 15–49, men aged 15 to 49 and children under 5, the survey aims to monitor and assess the health situation of the population. The sample design of the survey is meant to provide estimates on several health indicators at the national level, as well as at urban and rural areas and for fifteen and eighteen states of the country in 2010 and 2014surveys respectively. MICS applies stratified, two-stage cluster sampling techniques for data collection. In the first stage, clusters, also called enumeration areas (EA), based on the sampling frame of the recent national population census that is the 2008 census, are selected using probability proportional to size (PPS) [33, 34]. In the second stage, a fixed number of households (25 households per cluster) is selected from clusters selected in stage one using a systematic sampling technique. The detailed sampling methodology of the surveys has been described in detail in the respective surveys reports [33, 34].

### Variables and their measurements

Stunting is the primary variable of interest for the study. Stunting was measured as height-for-age (HAZ) less than minus 2 Standard Deviation (-2SD) from the median of the WHO child growth standard [10, 35]. For calculation of the percentage of under-five children that are stunted, the HAZ scores were recoded so that children whose HAZ falls between less than–2 SD and -6 SD from the WHO reference population are coded 1 and HAZ that lies between -2 SD and + 6 SD are coded as 0. The analysis was carried out on children who were born five years preceding the survey.

Inequality in childhood stunting was measured for five equity stratifiers. Economic status was approximated through a wealth index. The wealth index is customarily computed using durable goods, household characteristics and basic services, following the methodology explained elsewhere [36]. Though the type of asset variables used for constructing wealth index vary between surveys [37], the commonly used variables include water and sanitation facilities (WASH), radio, television, types of materials used to make the floor, roof, and wall of a household, car, bicycle, motorcycle, and electricity [36]. It has also been shown that any indicator or variable that is deemed important for indicating the economic status of households can be used in the construction of a wealth index [36]. The constructed wealth index is then divided into five quintiles: poorest (quintile 1), quintile 2, quintile 3, quintile 4, and richest (quintile 5). Maternal educational status was classified as no-education, primary education and secondary education or more, place of residence as urban versus rural. The sub-national region included the 13 regions in the country.

### Data analysis

As we briefly described in the data source sub-section above, the offline version of the WHO HEAT software updated in 2019 was used for analysis [38]. The burden of stunting was first disaggregated by the above-mentioned five equity stratifiers, i.e., economic status, education, place of residence, region, and sex. Following the disaggregation, stunting inequality was further analyzed using the four summary measures of health inequality: Difference (D), Ratio (R), Slope Index of Inequality (SII), and Relative Index of Inequality (RII). The choice of the summary measures for an inequality study should be based on the fact that the selected summary measures need to be of simple and complex measures [39]. At the same time, summary measures need to be relative and absolute measures to be able to examine inequality from different angles. For our study, we chose measures of inequality in accordance with this recommendation. While the D and R are simple measures, the SII and RII are complex measures [39]. Moreover, the D and SII are absolute measures, and the R and RII are relative measures. The simple measures of health inequality are used to compare health indicators (childhood stunting in our case) between two groups and are useful choices for dimensions of inequality, such as place of residence and sex. For dimensions of inequality with more than two categories such as wealth and education, however, more complex measures are required that account for the entire subpopulations in all the categories through simple measures can still be used. The detailed elucidation of the summary measures adopted in the present study has been clearly made elsewhere [38, 39]. Briefly, the difference (D) is a simple, unweighted measure of inequality that shows the absolute inequality between two subgroups. The ratio (R) is a simple, unweighted measure of inequality that shows the relative inequality between two subgroups. The slope index of inequality (SII) is a complex, weighted measure of inequality that represents the absolute difference in estimated values of a health indicator (childhood stunting) between the most-disadvantaged and most-advantaged while taking into consideration all the other subgroups–using an appropriate regression model. The relative index of inequality (RII) is a complex, weighted measure of inequality that represents the ratio of estimated values of a health indicator (childhood stunting) of the most-disadvantaged and most-advantaged to the health outcome indicators (childhood wasting) while taking into consideration all the other subgroups–using an appropriate regression model [38, 39].

The calculation of summary measures varied based on dimension inequality. That means it varied for ordered, non-ordered, and binary dimensions of inequality. D is calculated as the difference between two subgroups: for instance, for education, D was calculated as childhood stunting prevalence in the “uneducated” group, minus childhood stunting prevalence in the “secondary education” group. Economic status was calculated as childhood stunting prevalence in the poorest group, minus childhood stunting prevalence in the richest group. Similarly, D was calculated as childhood stunting prevalence in rural minus childhood stunting in urban populations with respect to the place of residence, male minus female for sex, and region with the highest estimate of childhood stunting minus the one with the lowest estimate of childhood stunting in relation to the subnational region. Except for dividing for ratio and minus for difference, the calculation and references are the same [38, 39].

To calculate SII, a weighted sample of the whole population is ranked from the most-disadvantaged subgroup (at rank zero or 0) to the most-advantaged subgroup (at rank 1). This ranking is weighted, accounting for the proportional distribution of the population within each subgroup. The population of each subgroup is then considered in terms of its range in the cumulative population distribution and the midpoint of this range. According to the definition currently used in HEAT, the health indicator of interest is then regressed against this midpoint value using a generalized linear model with a logit link, and the predicted values of childhood stunting are calculated for the two extremes (rank 1 and rank 0). For adverse health outcome indicators, such as childhood stunting, the SII value is calculated as the difference between the estimated values at rank 0 (v0) and rank 1 (v1) (covering the entire distribution):

$$\mathrm{SII}\:=\:\mathrm v0\textemdash\mathrm v1$$

To calculate RII, a weighted sample of the whole population is ranked from the most-disadvantaged subgroup (at rank zero or 0) to the most-advantaged subgroup (at rank 1). This ranking is weighted, accounting for the proportional distribution of the population within each subgroup. The population of each subgroup is then considered in terms of its range in the cumulative population distribution and the midpoint of this range. According to the definition currently used in HEAT, the health indicator of interest is then regressed against this midpoint value using a generalized linear model with logit link, and the predicted values of the health indicator are calculated for the two extremes (rank 1 and rank 0) [38, 39].

For adverse health outcome indicators, such as childhood stunting, the calculation is reversed and the RII value is calculated as the ratio of the estimated values at rank 0 (v0) to rank 1 (v1) (covering the entire distribution):

$$\mathrm{RII}\:=\:\mathrm v0\;/\;\mathrm v1$$

SII and RII are calculated for ordered dimensions. It is missing if at least one subgroup estimate or subgroup population share is missing [38, 39].

Regarding the interpretation of summary measures, if there is no inequality, D takes the value zero. Greater absolute values indicate higher levels of inequality. Positive values indicate a higher concentration of stunting among the disadvantaged and negative values indicate a higher concentration among the advantaged. If there is no inequality, R takes the value one. It takes only positive values (larger or smaller than 1). The further the value of R from 1, the higher the level of inequality. If there is no inequality, SII takes the value zero. Greater absolute values indicate higher levels of inequality. For adverse health outcome indicators, such as childhood stunting, positive values indicate a higher concentration of childhood stunting among the disadvantaged, and negative values indicate a higher concentration among the advantaged. If there is no inequality, RII takes the value one. RII takes only positive values, with values larger than one indicating a concentration of the indicator (childhood stunting) among the advantaged and values smaller than one indicating a concentration of childhood stunting among the disadvantaged. The further the value of RII from one, the higher the level of inequality [38, 39].

A 95% Confidence interval (CI) was computed to accompany the point estimates of stunting burden. As mentioned above, the CIs for Difference, SII, and Ratio and RII should not include 0 to conclude that there is inequality. On the other hand, the CIs for R should not contain 1 to declare the presence of stunting inequality between groups compared. Inequality trends were assessed in caution and by referring to the confidence intervals (CI) of each summary measure of different surveys. That means if the CIs didn’t overlap, there were increasing or decreasing changes, but the overlapping of CIs was considered a constant pattern. However, the small and large overlapping was not treated equally and authors considered this important concept during interpretations of trends. We followed similar procedures as previous inequality studies [10, 40]. To take care of the complex nature of the DHS’s and MICS data, all three design elements such as weight, cluster, and strata would be are taken into consideration during initial analysis by WHO inequality experts, that means, survey design specifications were taken into consideration during the analysis. The weight variable is v005 (or hv005 or mv005) divided by 1,000,000. The stratification is based on urban and rural areas in each region (v024 x v025). To apply the complex sample design parameters in estimating indicators each of the statistical software uses a different set of commands applying the sample design and producing the indicator estimates: Stata: svyset and svy: commands SPSS: csplan, csdescriptives and cstabulate commands R: survey package, including svydesign and other svy functions. The reasons for mixing unweighted/simple summary measures such as difference and ratio as well as weighted summary measures such as SII and RII are one indicator of authors' considered complex nature of DHSs or MICS data [38, 39].

### Ethical consideration

The analyses was done using the publicly available Health Equity Assessment Toolkit (HEAT) software. HEAT contains the disaggregated Multiple Indicator Cluster Survey (MICS) data that are publicly available via the WHO Health Equity Monitor database. HEAT has been cleared for dissemination and use by World Health Organization. Because the ethical clearance was approved by the institution that commissioned, funded and managed the overall MICS program, further ethical clearance was not required. Informed consent from the participants before survey was ensured by those responsible for survey deployment. The ICF International as well as an Institutional Review Board (IRB) in the country (Central Bureau of Statistics (Sudan)) also ensured that the protocols are in compliance with the U.S. Department of Health and Human Services regulations for the protection of human subjects.

## Results

In this study, a total of 23, 111 population (weighted) participated in both survey rounds. Of them, 11, 383 (49.2%) and 11, 005 (47.6%) were females and no educated subgroups respectively. About 16,558 (71.6%) and 5, 035 (21.7%) were rural residents and among the quintile one subgroup, respectively.

**Fig. 1:**
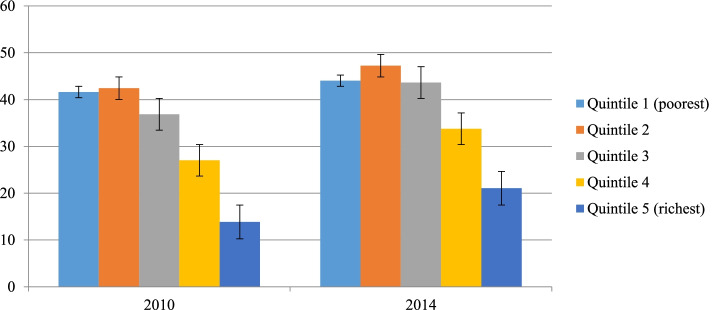
Childhood stunting across economic subgroups in Sudan from 2010 to 2014

The national average childhood stunting prevalence was 34.1% and 38.2% in 2010 and 2014, respectively. Childhood prevalence was significantly higher among the disadvantaged subgroups in both studied years. Regarding economic status, the prevalence in percentage points among the poorest subgroups in 2010 and 2014 was 41.6% and 44% respectively, whereas among the richest subgroups it was 13.8% and 21% in the same year, respectively. Except in quintile 1, which had a constant pattern, in all other economic subgroups, childhood stunting prevalence was significantly increasing over time (Fig. 1).

**Fig. 2:**
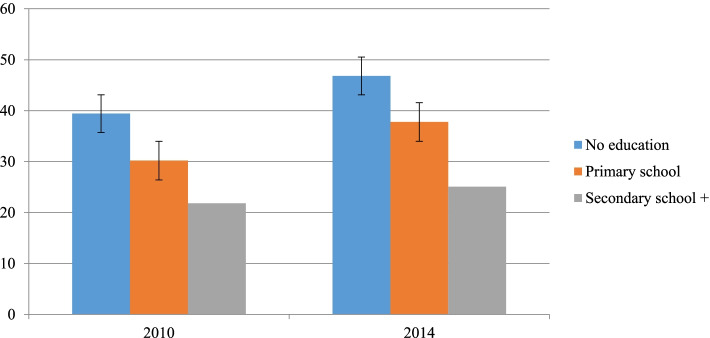
Childhood stunting across educational subgroups in Sudan from 2010 to 2014

The prevalence across educational subgroups was significantly different. For instance, the prevalence among no educated subgroups was 39.4 and 46.8 percentage points in 2010 and 2014 respectively, whereas among the secondary school and above subgroup it was 21.8 and 25 percentage points in the same survey years, respectively. Except in the secondary school and above subgroup, which had a constant pattern, among no educated and primary school subgroups, the pattern was significantly increasing over time (Fig. 2).

**Fig. 3:**
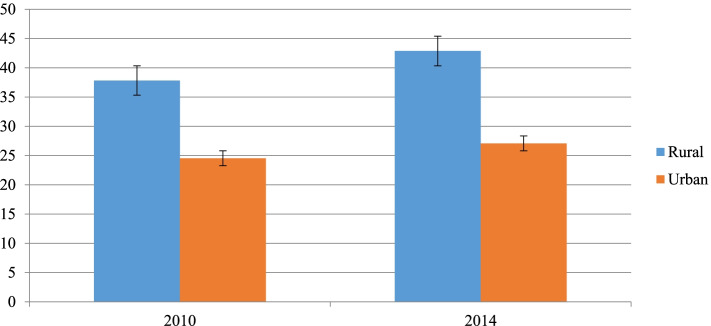
Childhood stunting by place of residence in Sudan from 2010 to 2014

In terms of residence, the result shows disparities with high prevalence among the rural residents. The pattern among rural residents was increasing over time, but it was constantly in the urban residents (Fig. 3).

**Fig. 4:**
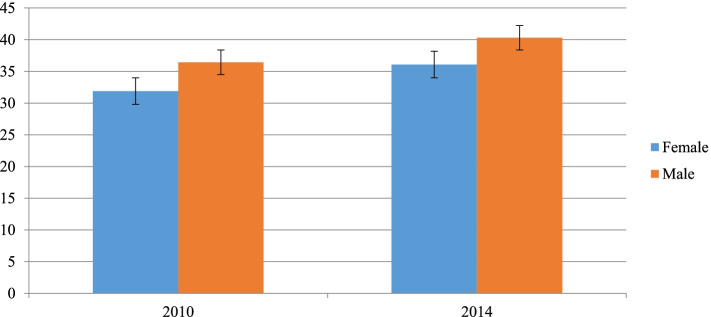
Childhood stunting by child sex in Sudan from 2010 to 2014

Similarly, the study shows a higher prevalence of childhood stunting among male children as compared to the females with an increasing pattern in both subgroups (Fig. 4).

Regional difference in the prevalence of childhood stunting was also observed in both survey years. For instance, in 2010, Red Sea, Kassala and Sinnar regions respectively were more affected, while Kassala, Central Darfur and Blue Nile respectively, were more affected regions in 2014. Some regions had increased and some of them had constant patterns (Table 1).

**Table 1:** Prevalence and overtime changes of childhood stunting across socio-economic, sex and area-based characteristics in Sudan from 2010 to 2014

Dimension of inequality	2010			2014	
	**Estimate (95%CI)**		**Pop**^**n**^	**Estimate (95%CI)**	**Pop**^**n**^
**Economic status**					
Quintile 1 (poorest)	41.61 (39.07, 44.19)		2909	44.03 (39.63, 48.53)	2126
Quintile 2	42.44 (40.27, 44.64)		2528	47.25 (44.38, 50.15)	2235
Quintile 3	36.86 (34.61, 39.16)		2491	43.64 (40.73, 46.58)	2481
Quintile 4	27.02 (24.54, 29.66)		2218	33.77 (30.84, 36.82)	2462
Quintile 5 (richest)	13.85 (11.60, 16.46)		1631	21.06 (17.64, 24.95)	2027
**Education**					
No education	39.42 (37.77, 41.11)		6501	46.82 (44.23, 49.43)	4504
Primary school	30.20 (28.17, 32.30)		3595	37.78 (35.61, 40.01)	4055
Secondary school +	21.81 (18.82, 25.14)		1595	25.08 (22.38, 27.99)	2760
**Residence**					
Rural	37.83 (36.30, 39.39)		8553	42.88 (40.83, 44.95)	8005
Urban	24.54 (21.92, 27.37)		3227	27.08 (24.46, 29.87)	3327
**Sex**					
Female	31.89 (30.30, 33.54)		5828	36.08 (34.09, 38.13)	5555
Male	36.44 (34.75, 38.17)		5951	40.31 (38.32, 42.34)	5777
**Region**					
Northern	Northern	23.21 (19.73, 27.09)	153	22.56 (18.07, 27.80)	208
River Nile	River Nile	29.81 (23.49, 37.02)	334	29.50 (23.92, 35.76)	335
Red Sea	Red Sea	51.95 (45.23, 58.59)	175	45.44 (37.87, 53.22)	178
Kassala	Kassala	48.16 (44.35, 51.99)	691	48.77 (41.20, 56.39)	400
Gadarif	Gadarif	39.14 (33.91, 44.64)	625	45.97 (39.57, 52.52)	657
Khartoum	Khartoum	21.67 (17.34, 26.72)	1702	21.86 (16.95, 27.73)	1593
Gezira	Gezira	28.41 (24.05, 33.21)	1579	41.59 (37.13, 46.18)	2045
Wite Nile	White Nile	35.59 (31.99, 39.36)	595	36.60 (31.44, 42.10)	561
Sinnar	Sinnar	46.04 (39.91, 52.29)	458	38.07 (32.19, 44.33)	465
Blue Nile	Blue Nile	36.72 (32.56, 41.10)	554	46.67 (42.15, 51.24)	655
North Kordofan	North Kordofan	45.52 (41.13, 49.98)	1195	40.80 (35.92, 45.86)	730
South Kordofan	South Kordofan^a^	35.83 (31.71, 40.18)	548	40.61 (36.38, 44.99)	413
North Darfur^a^	West Kordofan	34.42 929.46, 39.75) ^a^	878	42.53 (29.82, 56.32)	383
West Darfur^a^	North Darfor	35.98 (32.47, 39.64) ^a^	631	45.90 (41.45, 50.41)	758
South Darfur^a^	West Darfor	30.73 (27.16, 34.56) ^a^	1655	35.22 (27.72, 43.54)	217
NA	South Darfor	NA	NA	34.20 (27.47, 41.62)	1119
NA	Central Darfor	NA	NA	47.48 (40.89, 54.16)	156
NA	East Darfor	NA	NA	46.59 (41.66, 51.59)	452
National Average		34.1		38.2	

*NA* Not applicable since the region for 2010 survey are limited to fifteen, *indicate 2010 Region and their correspondence Prevalence, *Pop*^*n*^ population, *CI* Confidence Interval

### Socioeconomic and area-based inequality

**Fig. 5:**
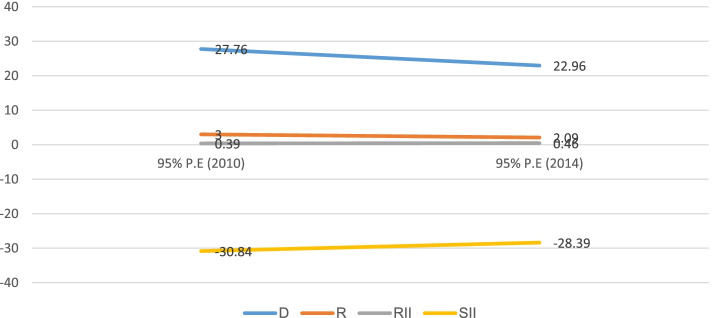
Economic based inequality in childhood stunting in Sudan from 2010 to 2014

Table 2 shows socioeconomic and area-based inequalities in childhood stunting in Sudan from 2010 to 2014. By all four inequality measures, the existence of economic inequality was observed. For instance, by the Difference measure, the economic-based inequality was 27.7% (95% CI; 24.23%, 31.28%) and 22.9% (95% CI; 17.20%, 28.72%) in 2010 and 2014, respectively. Economic-based inequality was observed in childhood stunting with higher concentration among the poorest subgroups, and the overtime change of economic inequality was constant. Similarly, the economic inequality by SII measure in 2010 (-30.8%, 95% CI; -33.68%, -27.99%) and 2014 (-28.3%, 95% CI; -31.37%, -25.40%), indicates higher concentration of childhood stunting in the disadvantaged subgroups (poorest). Overlapping of intervals in both surveys by SII measure also confirmed that overtime change of economic inequality was constant (Fig. 5).

**Table 2:** Trends of socio-economic inequality in childhood stunting in Sudan: evidence from Sudan multiple indicator cluster surveys (2010–2014)

Dimension of Inequalities	Measures of inequalities	2010	2014
		**%(95%CI)**	**%(95%CI)**
Economic status	D	27.76 (24.23, 31.28)	22.96 (17.20, 28.72)
	R	3.00 (2.44, 3.56)	2.09 (1.67, 2.50)
	RII	0.39 (0.15, 0.62)	0.46 (0.28, 0.64)
	SII	-30.84 (-33.68, -27.99)	-28.39 (-31.37, -25.40)
Education	D	17.60 (14.04, 21.17)	21.73 (17.91, 25.55)
	R	1.80 (1.53, 2.07)	1.86 (1.63, 2.09)
	RII	0.47 (0.26, 0.68)	0.43 (0.23, 0.64)
	SII	-24.84 (-28.09, -21.59)	-30.47 (-33.57, -27.38)
Place of residence	D	13.28 (10.15, 16.41)	15.80 (12.40, 19.19)
	R	1.54 (1.35, 1.72)	1.58 (1.40, 1.75)
Sex	D	4.54 (2.19, 6.89)	4.22 (1.38, 7.07)
	R	1.14 (1.06, 1.22)	1.11 (1.03, 1.20)
Region	D	30.27 (22.09, 38.45)	26.90 (17.55, 36.25)
	R	2.39 (1.79, 3.00)	2.23 (1.57, 2.88)

**Fig. 6:**
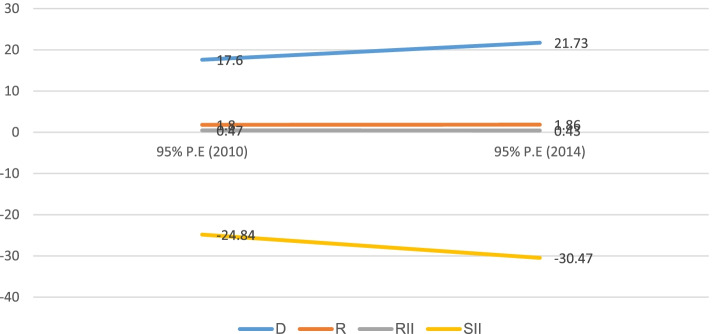
Education-based inequality in childhood stunting in Sudan from 2010 to 2014

The finding also shows significant education-based inequality in childhood stunting in Sudan from 2010 to 2014 by all four summary measures. For instance, based on the Ratio measure the education-based inequality was seen both in 2010 (1.8%, 95% CI; 1.53%, 2.07%) and 2014 (1.8%, 95% CI; 1.63%, 2.09%) survey periods, with more concentration of stunting burden among the non-educated subgroups. Education-based inequality was constant over time using simple summary measures (Difference and Ratio). Similarly, the education-based inequality was also seen using SII, both in 2010 (-24.8%, 95% CI; -28.09%, -21.59%) and 2014 (-30.4%, 95% CI; -33.57%, -27.38%) with higher burden among the disadvantaged subgroups (no educated) as compared to subgroups who attend secondary and above schools (Fig. 6).

**Fig. 7:**
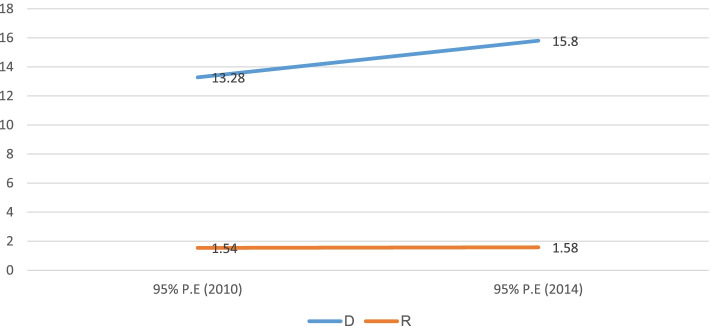
Urban–rural inequality in childhood stunting in Sudan from 2010 to 2014

In terms of place of residence inequality, the finding shows both absolute and relative inequality in childhood stunting in both of the studied survey periods. For instance, the Difference measure 13.2%, 95% CI; 10.15%, 16.41% in 2010 and 15.8%, 95% CI; 12.40%, 19.19% in 2014 shows the place of residence inequality in childhood stunting with more burden among rural residents and with no change between 2010 and 2014 (Fig. 7).

**Fig. 8:**
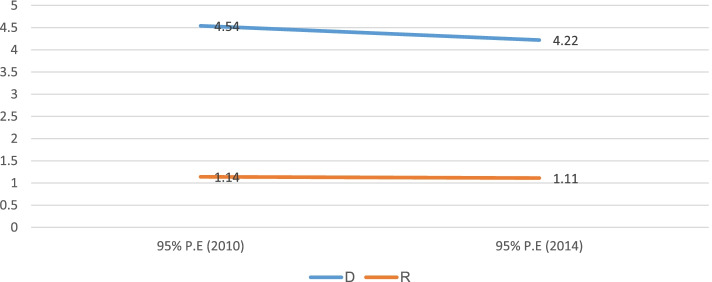
Sex-related inequality in childhood stunting in Sudan from 2010 to 2014

The finding from this study also demonstrated that there was sex-related inequality in childhood stunting in Sudan from 2010 to 2014. For instance, using the Difference measure, sex-related inequality was seen in 2010 (4.5%, 95% CI; 2.19%, 6.89%) and 2014 (4.2%, 95% CI; 1.38%, 7.07%), with more burden among male children, but no overtime change between the two studied survey periods (Fig. 8).

**Fig. 9:**
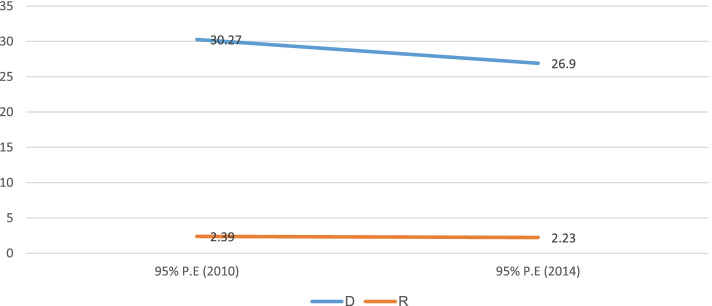
Subnational region inequality in childhood stunting in Sudan from 2010 to 2014

Another main finding from this study was regional inequality in childhood stunting in both surveys. For instance, based on the Ratio measure, the regional inequality was seen in 2010 (2.3%, 95% CI; 1.79%, 3.00%) and 2014 (2.2%, 95% CI; 1.57%, 2.88%) with no significant change between the 2010 and 2014 survey periods (Fig. 9).

Table 2 shows detail about the extent and overtime changes of socio-economic and area-based inequality in Sudan in both survey years (Table 2).

## Discussion

In this study, we attempted to investigate the extent as well as the overtime dynamics of stunting in children in Republic of Sudan. For this purpose, we relied on the nationally representative data from Sudan multiple indicator cluster survey, collected between 2010 and 2014. In general, the overall average childhood stunting in this study increased from 34.1% in 2010 to 38.2% in 2014. This steady increment was opposite to the global target to reduce under-five child stunting [27]. This may be characterized by an increased rate of poverty in Sudan [41] or inequitable resource-sharing and governance [25].

In Sudan, hunger is becoming more likely when food costs rise and inflation rises [42]. The World Food Program (WFP) estimates that 5.8 million people in Sudan are food insecure [43]. Sudanese people typically struggle to acquire enough food due to their low purchasing power [42]. A typical local food basket, for example, costs at least three-quarters of a Sudanese household's income [42]. Additionally, food insecurity is exacerbated by Sudan's unpredictable economy. The instability stems from a lack of infrastructure and the loss of a substantial portion of oil money following South Sudan's secession. Sudan has been trying to recover from these setbacks for quite some time now [42].

Sudanese children suffer from severe malnutrition and stunting. Hunger causes malnutrition and stunting, or a reduction in growth. More than half a million Sudanese youngsters are critically malnourished [44]. Furthermore, more than a third of children under the age of five, or 2.3 million, are stunted [45]. Sudan is primarily reliant on the fragile agricultural industry. This reduces food security and increases hunger in Sudan, particularly because it employs 80 percent of the country's workforce [42]. Agriculture is unreliable due to a number of variables. Sudan is subjected to natural disruptions such as desertification, droughts, and floods on a regular basis. It also faces water contamination and a lack of adequate water supply [42]. Moreover, many Sudanese displaced people are at risk of starvation. Internal displacement of approximately two million Sudanese has resulted from ongoing domestic crises in the country [46]. In addition, more than a million refugees have arrived, the majority of whom are from South Sudan. Internally displaced people and refugees frequently rely on food aid [42].

Similar higher prevalence stunting was observed in Burundi, which has been facing political instabilities [18]. But, Tanzania, in the same region, had a declining trend of stunting explained by political commitment and increased agricultural production, important for child nutrition and growth [18]. The burden of stunting based on our study was the worst among the disadvantaged subgroups, namely the poorest, uneducated, Kassala and Central Darfur region of Sudan in both studied survey. Being a male child was also one dimension of being unequally affected by stunting.

Our findings confirmed that both absolute and relative economic inequalities in childhood stunting were stark in both household surveys spaced by five years. The poorest subgroup of the population was hugely affected by stunting than richest. Congruent to our findings, studies done previously confirmed that inequality existed in the nutritional status among a subgroup of the population, with the poorest subgroup endured the highest stunting rates [22, 47–50]. This may be due partly to the fact that food insecurity due to drought and underdevelopment commonly affected the rural poor residents of Sudan [51]. The other possible explanation contributed to the socioeconomic inequality in stunting may be attributed to the low dietary intake among the poor children [48] or marginalized from access to health care by poor [25] and mother’s attainment of higher education [50]. Drought resistance seed production strategy is an appropriate intervention to avoid food insecurity faced the poor due to drought [52]. Improve political commitment to tackle under nutrition [53] facing the poor through increasing income and reducing poverty [54]. The relative measure of economic inequality of stunting between the two survey periods decreased. It can be characterized by further deterioration in the living conditions of families over the years due to recurrent conflicts and high food prices in Sudan [55] or reduced revenue from oil export [55] for the richest subgroup of the population.

We found disproportionally affected under five children by stunting from uneducated subgroup population of our study done on the two survey periods in Sudan. This finding was consistent with the reports of previous studies that revealed the presence of significant discrepancy in the proportion of stunted children that disfavor the uneducated categories of the population [21, 56, 57]. Since 1990, Sudanese government started to enhance education by eradicating illiteracy among its population [41]. However, there was limited access to education in the rural parts of Sudan [58]. Studies confirmed that parent’s education promotes child nutrition either by improving acquisition of health knowledge or adherence to recommended feeding practices for children [59]. Difference measure of an inequality for education variable increased during the study period. This may directly linked with high resource allocation to security sectors in-expense of social sectors, like accessing education to everyone [60]. Reducing inequality to achieve goal 10 of SDGs [61] and equity may be assured if future education policy of Sudan focused on reaching those marginalized to education.

Our analysis of both Sudan demographic and health survey showed that there was a marked inequality existed in stunting among rural and urban residents. Children lived in the rural area carried more burden of stunting in comparison to those lived in urban area. This was consistent with previous studies that evidenced inequality of childhood stunting across rural and urban residence [21, 51, 62]. This may be due to the difference in income and poverty between urban and rural setting [63, 64] and access to health care or rural illiteracy [63]. Overtime disparity with regard to residence showed no change between the two survey periods. This may be due to lack of policy action targeting the disadvantaged rural area communities either to increase their income or educational status [65] or the gap of capacities of health facilities [66]. It may be better to have developed rural agricultural policies that have a positive effect for income generation to community in the rural area [67]. An effort that focus for the provision of essential infrastructure and access to education in rural setting can narrow the observed inequality [64].

We revealed a wide disparity in stunting between male and female children, both in absolute and relative measure. This result agrees with the study done in three middle-income African countries that stunting rates higher among male children than female children [21, 22]. This may due to male child’s repeated vulnerability to diarrhea or any other morbidity than female child [67] as diarrhea is one of the determinants of stunting [68, 69]. No change was seen with regard to sex variable for measurement of inequality over the study period.

The disparity in childhood stunting within subpopulations disproportionately affects the geopolitical zone of Kassala, Red Sea and Central Darfur region. Regional disparity trends in stunting rates were preserved over this time period. The possible explanation may be unevenly distributed governmental public spending for region affected by displacement and insecurity due to civil war in the country [60]. The other may be due to unequal wealth distribution along the regional lines, especially the agriculture part irrigation sector [60, 70]. The low effective use of health services due to limited geographic access and the continuous influx of displaced populations and refugees in the region highly affected by stunting [66] may also characterize the observed inequality. Policy efforts that prioritize the region greatly affected by stunting can narrow equity gaps in stunting burden among marginalized region and this is the responsibility of influential decision makers.

The strength of the study was that it used nationally collected data for analysis. To our best knowledge, this is the first study done considering geographic and socioeconomic inequality of stunting in Sudan that has input to policy makers. The study has the following limitation. First, as it was analyze data collected through cross-sectional method, it is difficult to know cause and effect of inequality of stunting during childhood period. Second, the study did not answer why inequality existed among the subpopulation category of Sudan. We suggested further studies need to be done that can help to identify the cause of inequality in the population using decomposition analysis. 

## Conclusion

The magnitude of stunting was increased nationally over time. While sex, residence and geographic related inequalities remain unchanged over time, economic status and educational inequality had seen a change by some inequality measure over the same time period. The inequality disproportionally affected children from poorest quintile, children from uneducated parents, being male child, has been living in the rural area and in the region of Kassala, Red Sea and Central Darfur.

We recommend the nation to work aggressively to reduce poverty that commonly affects the poorest and those living in the rural areas by investing in crop based agricultural sector. The implementation nutritional policy of the country should be also given more attention to those in the rural and region repeatedly affected by conflict and displacement. Though the government works for universal education, there is yet to address peoples without education. The issue of malnutrition is complex and should be the responsibility of each governmental cabinet to narrow the existed inequality of stunting in Sudan by developing multi-sectored strategy.

## Data Availability

The datasets generated and/or analyzed during the current study are available in the WHO’s HEAT version 3.1 [https://www.who.int/gho/health_equity/assessment_toolkit/en/].

## Abbreviations

CI: Confidence Interval
D: Difference
DHS: Demographic and Health Survey
EA: Enumeration Area
HAZ: Height-for-Age
HEAT: Health Equity Assessment Toolkit
LMICs: Low-and Middle Income Countries
MICS: Multiple Indicator Cluster survey
SII: Slope Index of Inequality
RII: Relative Index of Inequality
R: Ratio
SDG: Sustainable Development Goal
WHO: World Health organization

## References

[1] Beal T, Tumilowicz A, Sutrisna A, Izwardy D, Neufeld LM (2018). A review of child stunting determinants in Indonesia. Matern Child Nutr.

[2] Aguilera Vasquez N, Daher J (2019). Do nutrition and cash-based interventions and policies aimed at reducing stunting have an impact on economic development of low-and-middle-income countries? A systematic review. BMC Public Health.

[3] Angdembe MR, Dulal BP, Bhattarai K, Karn S (2019). Trends and predictors of inequality in childhood stunting in Nepal from 1996 to 2016. Int J Equity Health.

[4] Hossain M, Choudhury N, Khaleda Adib Binte Abdullah,etal. Evidence-based approaches to childhood stunting in low and middle income countries:a systematic review. Arch Dis Child 2017; 102:903–909. 10.1136/archdischild-2016-311050 PMC5739821 28468870

[5] De Onis M, Branca F. Childhood stunting: a global perspective. Matern Child Nutr. 2016; 12 Suppl 1(Suppl 1):12–26. 10.1111/mcn.12231 PMC5084763 27187907

[6] Binagwaho A, Rukundo A, Powers S, Donahoe KB, Agbonyitor M, Ngabo F, et al. Trends in burden and risk factors associated with childhood stunting in Rwanda from 2000 to 2015: policy and program implications. BMC Public Health 2020; 20; 20(1):83. 10.1186/s12889-020-8164-4 PMC6971879 31959142

[7] Aguayo VM, Menon P (2016). Stop stunting: improving child feeding, women’s nutrition and household sanitation in South Asia. Matern Child Nutr.

[8] Menon P, Headey D, Avula R, Nguyen PH (2018). Understanding the geographical burden of stunting in India: Aregression-decomposition analysis of district-level data from 2015–16. Matern Child Nutr.

[9] María Clara Restrepo-Méndez, Aluísio JD Barros1, Robert E Black and Cesar G Victora. Time trends in socio-economic inequalities in stunting prevalence: analyses of repeated national surveys. Public Health Nutrition. 2097–2104;18:12. 10.1017/S1368980014002924 PMC4909139 25521530

[10] Shibre G, Zegeye B, Haidar J (2020). Extent of and trends in inequalities in child stunting in Sierra-Leone from 2005 to 2013: evidence from demographic and healthsurveys and multiple indicator cluster surveys. International Journal for Equity in Health.

[11] Jomah JEAES. Determinants of Child Malnutrition in Sudan. International Journal of Multidisciplinary Approach and Studies.2018; 5(2).

[12] World Health Organization. Regional Office for the Eastern Mediterranean. (2013). Saving the lives of mothers and children: rising to the challenge in South Sudan. Available at: https://apps.who.int/iris/handle/10665/116149

[13] Andrew M. Prentice Stunting in Developing Countries. Nutrition and Growth.2018; (117): 165–175. Accessed on Dec 29, 2021. 10.1159/000484505 29393119

[14] Emmanuel S, Katja V, Ryoko S. All Hands on Deck: Reducing Stunting through Multisectoral Efforts in Sub-Saharan Africa. Africa Development Forum; Washington, DC: World Bank and Agence française de développement. 2019 © World Bank. https://openknowledge.worldbank.org/handle/10986/32037. License: CC BY 3.0 IGO.

[15] Lu C, Cuartas J, Fink G, McCoy D, Liu K, Li Z (2020). Inequalities in early childhood care and development in low/middle-income countries: 2010–2018. BMJ Glob Health.

[16] Kismul H, Acharya P, Mapatano MA, Hatløy A (2017). Determinants of childhood stunting in the Democratic Republic of Congo: further analysis of Demographic and Health Survey 2013–14. BMC Public Health.

[17] da Silva ICM, França GV, Barros AJD, Amouzou A, Krasevec J, Victora CG (2018). Socioeconomic Inequalities Persist Despite Declining Stunting Prevalence in Low- and Middle-Income Countries. J Nutr.

[18] Musheiguza E, Mahande MJ, Malamala E, Msuya SE, Charles F, Philemon R (2021). Inequalities in stunting among under-five children in Tanzania: decomposing the concentration indexes using demographic health surveys from 2004/5 to 2015/6. Int J Equity Health.

[19] Ebaidalla EM. Understanding Inequality ofOpportunity in Child Health in Sudan.2019. Available at: https://igmozambique.wider.unu.edu/sites/default/files/Event/Ebaidalla%20MAHJOUB%20AHMED.pdf. Accessed on June 1, 2021

[20] Iftikhar A, Bari A, Bano I, Masood Q (2017). Impact of maternal education, employment and family size on nutritional status of children. Pak J Med Sci.

[21] WHO. Reducing stunting in children: equity considerations for achieving the Global Nutrition Targets 2025.2018. Available at: https://www.who.int/publications/i/item/9789241513647. Accessed on June 1, 2021

[22] Jonah CMP, Sambu WC, May JD (2018). A comparative analysis of socioeconomic inequities in stunting: a case of three middle-income African countries. Arch Public Health.

[23] Novignon J, Aboagye E, Agyemang OS, Aryeetey G. Socioeconomic-related inequalities in child malnutrition: evidence from the Ghana multiple indicator cluster survey. Heal Econ Rev. 2015;5:34. 10.1186/s13561-015-0072-4 PMC4658346 26603158

[24] Ekholuenetale M, Barrow A, Ekholuenetale CE, Tudeme G (2020). Impact of stunting on early childhood cognitive development in Benin: evidencerom Demographic and Health Survey. Egyptian Pediatric Association Gazette.

[25] Baye K, Laillou A, Chitweke S (2020). Socio-Economic Inequalities in Child Stunting Reduction in Sub-Saharan Africa. Nutrients.

[26] European Union. The social and economic consequences of malnutrition in ACP countries. Available at: https://www.europarl.europa.eu/meetdocs/2009_2014/documents/acp/dv/background_/background_en.pdf. Accessed on June 1, 2021

[27] de Onis M, Dewey KG, Borghi E, Onyango AW, Blössner M, Daelmans B, Piwoz E, Branca F. The World Health Organization's global target for reducing childhood stunting by 2025: rationale and proposed actions. Matern Child Nutr. 2013 Sep; 9 Suppl 2(Suppl 2):6–26. 10.1111/mcn.12075 PMC6860845 24074315

[28] UN. Transforming our world: the 2030 Agenda for Sustainable Development.2015. Available at: https://sustainabledevelopment.un.org/post2015/transformingourworld/publication. Accessed on June 1, 2021

[29] WHO. Global Health Observatory (GHO) data. Health Equity Assessment Toolkit.Overview.2020. Available from: https://www.who.int/gho/health_equity/assessment_toolkit/en/. Accessed on Dec 29, 2021

[30] Hosseinpoor AR, Nambiar D, Schlotheuber A, Victora C, Boerma T, Barros AJD. Health Equity Assessment Toolkit (HEAT): software for exploring and comparing health inequalities in countries. BMC Med Res Methodol. 2016. 10.1186/s12874-016-0229-9 PMC5069829 27760520

[31] Hosseinpoor AR, Bergen N, Schlotheuber A, Victora C, Boerma T, Barros AJD (2016). Data Resource Profile: WHO Health Equity Monitor (HEM). Int J Epidemiol.

[32] The World Bank. Multiple Indicator Cluster Survey 2014.2021. Available at: https://microdata.worldbank.org/index.php/catalog/2656. Accessed on June 1, 2021

[33] Central Bureau of Statistics (CBS), UNICEF Sudan. 2016, Multiple Indicator Cluster Survey 2014 of Sudan, Final Report. Khartoum, Sudan: UNICEF and Central Bureau of Statistics (CBS), February 2016.

[34] The World Bank. Household Health Survey 2010.2021. [Available at: https://microdata.worldbank.org/index.php/catalog. Accessed on June 1, 2021

[35] WHO.WHO Multicentre Growth Reference Study Group. WHO Child Growth Standards: Length/height-for-age, weight-forage, weight-for-length, weight-for-height and body mass index-for-age: methods and development. WHO, Geneva, 2006 .2020. Available at: https://www.who.int/publications/i/item/924154693X. Accessed on June 1, 2021

[36] Rutstein SO, Johnson K. The DHS wealth index, 2004. Available at: http://www.measuredhs.com/pubs/pdf/CR6/CR6. Accessed on June 1, 2021

[37] Martel P. Review of options for reporting water, sanitation and hygiene coverage by wealth quintile, MICS Methodological Papers, No. 4, Data and Analytics Section, Division of Data, Research and Policy, UNICEF New York. 2016.

[38] WHO. Health Equity Assessment Toolkit (HEAT): Software for exploring and comparing health inequalities in countries. Builtin database edition.Version 3.1. Geneva: World Health Organization; 2019.

[39] WHO. Handbook on health inequality monitoring with a special focus on low and middle income countries. Geneva: World Health Organization; 2013.

[40] Zegeye B, Shibre G, Idriss-Wheeler D, Yaya S. Trends in inequalities in childhood stunting in Ethiopia from 2000 to 2016: a cross sectional study. J Public Health (Oxf). 2020:fdaa051. 10.1093/pubmed/fdaa051 32424422

[41] Mohsin MIA. REVIEW OF THE SUDANESE SOCIOE-CONOMIC DEVELOPMENT PROBLEMS.Journal of Economic Cooperation 2002; 23(3):85–102.

[42] Borgen Project. Available at: https://borgenproject.org/5-facts-about-hunger-in-sudan/. Accessed on Dec 29, 2021.

[43] Carnegie Endowment. Available at: https://carnegieendowment.org/2012/05/16/sudan-from-conflict-to-conflict-pub-48140. Accessed on Dec 29, 2021.

[44] World Food Program. Available at: https://www.wfp.org/countries/sudan. Accessed on Dec 29, 2021.

[45] UNICEF. Available at: https://www.unicef.org/sudan/health-nutrition. Accessed on Dec 29, 2021.

[46] USAID. Available at: https://www.usaid.gov/sudan/food-assistance. Accessed on Dec 29, 2021.

[47] Nwosu CO, Ataguba JE. Explaining changes in wealth inequalities in child health: The case of stunting and wasting in Nigeria. PLoS One. 2020; 15(9):e0238191. 10.1371/journal.pone.0238191 PMC7489558 32925960

[48] Mohammed SH, Muhammad F, Pakzad R, Alizadeh S. Socioeconomic inequality in stunting among under-5 children in Ethiopia: a decomposition analysis. BMC Res Notes 12, 184. 10.1186/s13104-019-4229-9 PMC6440115 30922416

[49] Kien VD, Lee HY, Nam YS, Oh J, Giang KB, Van Minh H (2016). Trends in socioeconomic inequalities in child malnutrition in Vietnam: findings from the Multiple Indicator Cluster Surveys, 2000–2011. Glob Health Action.

[50] Krishna A, Mejía-Guevara I, McGovern M, Aguayo VM, Subramanian SV. Trends in inequalities in child stunting in South Asia. Matern Child Nutr. 2018; 14 Suppl 4(Suppl 4):e12517. 10.1111/mcn.12517 PMC6519254 29048726

[51] Osama S. Nutrition Country Profile, the Republic of the Sudan, 2005. Available at: https://www.researchgate.net/publication/265698534_Nutrition_Country_Profile_the_Republic_of_the_Sudan/citation/download. Accessed on June 2, 2021

[52] National Council Combating Desertification and UN. REPUBLIC OF SUDAN National Council for Combating Desertification (NCCD): Sudan National Drought Plan.2018.

[53] WFP and UNICEF. The Case for Investment in Nutrition in Sudan.2014.

[54] Fatima K, Awadia A, Siteldar A, Zemzem Y, Sara S, Tahani S, et al. Households dietary habits and food consumption patterns in Hamashkoreib locality, Kassala State, Sudan. Journal of Ethnic Foods. 2017; 4. 10.1016/j.jef.2017.08.009.

[55] FAO. AQUASTAT Country Profile – Sudan. Food and Agriculture Organization of the United Nations (FAO). Rome, Italy.2105

[56] Garcia S, Sarmiento OL, Forde I, Velasco T (2013). Socio-economic inequalities in malnutrition among children and adolescents in Colombia: the role of individual-, household- and community-level characteristics. Public Health Nutr.

[57] Apio BRS, Mawa R, Lawoko S, Sharma KN (2019). Socio-economic Inequality in Stunting among Children Aged 6–59 Months in a Ugandan Population Based Cross-sectional Study. American Journal of Pediatrics.

[58] Sudan House Hold Health Survey Second Round 2010: Summary Report .2011. Available at: https://reliefweb.int/report/sudan/sudan-household-and-health-survey-second-round-2010-summary-report. Accessed on June 1, 2021

[59] Huda TM, Hayes A, El Arifeen S, Dibley MJ (2018). Social determinants of inequalities in child undernutrition in Bangladesh: A decomposition analysis. Matern Child Nutr.

[60] African Economic Outlook. Sudan 2012.

[61] World Social Report 2020: Inequality in a Rapidly Changing World. United Nations Department of Economic and Social Affairs 2020. Available at: https://www.un.org/development/desa/dspd/world-social-report/2020-2.html. Accessed on June 1, 2021

[62] Akombi BJ, Agh KE, Renzaho AM, Hall JI, Merom DR (2019). Trends in socioeconomic inequalities in child undernutrition: Evidence from Nigeria Demographic and Health Survey (2003–2013). PLoS ONE.

[63] Zere E, McIntyre D (2003). Inequities in under-five child malnutrition in South Africa. Int J Equity Health.

[64] IFAD. Enabling the rural poor to overcome poverty in Sudan.2007. Available at: https://www.ifad.org/documents/38714170/39309787/eng_1.pdf/69805352-18ca-47b2-82c7-4848121337cb. Accessed on June 1, 2021

[65] UNICEF. Sudan Country programme document 2013–2016. Available at: https://digitallibrary.un.org/record/730846?ln=en. Accessed on June 1, 2021

[66] Central Bureau of Statistics (CBS), UNICEF Sudan. Multiple Indicator Cluster Survey 2014 of Sudan, Final Report. Khartoum, Sudan: UNICEF and Central Bureau of Statistics (CBS). 2016.

[67] Bertolini P. OVERVIEW OF INCOME AND NON-INCOME RURAL POVERTY IN DEVELOPED COUNTRIES.2019. Available at: https://www.un.org/development/desa/dspd/wp-content/uploads/sites/22/2019/03/bertolini-presentation-on-rural-poverty-developed-countries-2.pdf. Accessed on June 1, 2021

[68] Hung BV. The most common causes of and risk factors fordiarrhea among children less than five years of age admittedto Dong Anh Hospital, Hanoi, Northern Vietnam.2006.

[69] Yang YY, Kaddu G, Ngendahimana D, Barkoukis H, Freedman D, Lubaale YA (2018). Trends and determinants of stunting among under-5s: evidence from the 1995, 2001, 2006 and 2011 Uganda Demographic and Health Surveys. Public Health Nutr.

[70] Omer AM (2011). Agriculture Policy in Sudan. Agricultural Science Research Journal.

